# The Centre for International Mental Health Approach to Mental Health System Development

**DOI:** 10.3109/10673229.2012.649090

**Published:** 2012-02-15

**Authors:** Harry Minas

**Affiliations:** From the Centre for International Mental Health, Melbourne School of Population Health, University of Melbourne

**Keywords:** development, leadership, mental health systems, scaling up

## Abstract

Although mental disorders are a major public health problem, the development of mental health services has been a low priority everywhere, particularly in low- and middle-income countries. Recent years have seen a growing understanding of the importance of population mental health and increased attention to the need to developmental health systems for responding to population mental health service needs. In countries and regions where mental health services are all but nonexistent, and in postconflict and postdisaster settings, there are many impediments to establishing or scaling up mental health services. It is frequently necessary to act simultaneously on multiple fronts: generating local evidence that will inform decision makers; developing a policy framework; securing investment; determining the most appropriate service model for the context; training and supporting mental health workers; establishing or expanding existing services; putting in place systems for monitoring and evaluation; and strengthening leadership and governance capabilities. This article presents the approach of the Centre for International Mental Health in the Melbourne School of Population Health to mental health system development, and illustrates the way in which the elements of the program are integrated by giving a brief case example from Sri Lanka. (harv rev psychiatry 2012;20:37–46.)

Mental disorders are a major public health problem, and the biggest public health problem among young people in the most productive years of life. Taken together, the high prevalence of mental disorders,^1^ the annual loss of life from suicide (>300,000/year in Asia,^2^ the most common cause of death among young adults), the decreased longevity of people with schizophrenia (life expectancy 15–20 years less than the general population), the high disability burden attributable to mental disorders (>30/ of all disability-adjusted life years from noncommunica-ble diseases),^3^ the massive loss of economic productivity, and the abject poverty^4^ and misery of so many people with mental disorders (most of whom have no access to treatment and care in low- and middle-income countries (LAMICs)) suggest the dimensions of the problem. And yet mental illness, along with the health systems that need to be developed to provide adequate treatment and care and to improve population mental health, has been largely ignored by governments, bilateral aid agencies, other major development funders, nongovernmental organizations (NGOs) promoting international development, researchers, and educators.^5^

The key mental health system issues and challenges in LAMICs are now well known^6^ (see text box next page). Mental health is still a low priority in most LAMICs and therefore attracts little government attention^7^ and weak investment.^8^,^9^ There are low levels of mental health literacy in populations and also low population demand for services. There is therefore both little investment in developing mental health services and a shortage of everything necessary—skilled workers, health facilities, and drugs.^9^–^11^ The available mental health workforce is poorly distributed and, in many places, almost entirely limited to large urban centers and mental hospitals.^12^–^14^ The great majority of the population has little or no access to mental health assessment, treatment, and care.^15^,^16^ In culturally diverse populations, services are typically provided only in the dominant language and culture. Neglect and abuse of people with mental illness are frequently found in mental hospitals, in social institutions for the mentally ill homeless and destitute,^17^ and in the community.^18^ Stigma, discrimination, and human rights abuses are ubiquitous^19^ and will require concerted efforts to eliminate.^19^–^21^ The frequently extreme poverty of the mentally ill results from the lack of free and accessible mental health services, coupled with social and economic exclusion.^4^

**Table:** 

Mental Health System Issues and Challenges		
Context	System elements	Outcomes
• Little understanding of mental health as an important public health and social and economic development issue	• Inadequate infrastructure, facilities, equipment, drug distribution systems	• Narrow population coverage: wide “treatment gap”
• Little understanding that effective and affordable interventions and service models are available	• Shortage of skilled mental health workers	• Very wide gap between best (usually in major urban centers) and worst (usually in poor rural areas) mental health services
• Mental health is a low political and social priority	• Geographic maldistribution of available workforce	• Low and inequitable access (geographic, economic, linguistic, cultural) to mental health services
• Weak investment	• Disciplinary imbalance: dominated by physicians and nurses	• Stigma, discrimination, social and economic exclusion
• Weak drive for mental health system reform and development	• Hospital centered	• Mental health training is unattractive for most disciplines
• Low levels of skill in policy development and implementation	• Undeveloped information systems, with lack of high-quality local information to support planning	• Inadequate protection of rights, with widespread human rights abuses
• Weak governance and management arrangements	• Poorly developed mental health systems research capacity	• Lack of locally relevant evidence for policy and practice
• Low population “mental health literacy”	• No culture of evaluation or continuous quality improvement	• Poorly developed advocacy by civil society and groups
	• Poorly organized and marginalized consumers, carers, civil society	

## MOMENTUM FOR CHANGE

While effective mental health services are unavailable for most people in LAMICs, there is a renewed commitment to focus attention on the mental health of populations^5^ and on the scaling up of mental health services that have the capacity to respond to mental health service needs.^22^,^23^

In August 2007, the *International Journal for Mental Health Systems* (see below) was launched with the intention of focusing attention on mental health system development.^5^ In September 2007, the *Lancet* published a series of papers that set out the current mental health situation in LAMICs and that proposed strategies for scaling up mental health services.^7^,^9^,^10^,^15^,^22^,^24^ The *Lancet*'s editor suggested that there was a need to launch a new movement for global mental health.^25^ In 2008, following publication of a review of developments one year after the *Lancet* series,^26^ the Movement for Global Mental Health was formally launched,^6^ with the aim of “improv[ing] services for people with mental disorders worldwide through the coordinated action of a global network of individuals and institutions.”^27^ In October 2008, the World Health Organization (WHO) Mental Health Gap Action Programme^31^ (mh-GAP) was launched. The intent of mhGAP is to scale up care for mental, neurological, and substance use disorders, with strategies identified particularly for resource-constrained settings and countries. In October 2011, the *Lancet* published a second series^4^,^11^,^19^,^28^–^30^ and reaffirmed its commitment to global mental health.^23^

Mental health system development is making its way onto the agendas of bilateral development agencies and international development NGOs. The Australian Agency for International Development (AusAID) *Development for All*^32^ disability strategy is a substantial achievement in promoting disability-inclusive development practice across AusAID programs. The strategy may open the way for increased attention to mental disorders, the single most important contributor to disability in LAMICs. In his statement of support for the WHO publication *Mental Health and Development*, the former Australian minister for international development noted that

Australia is committed to reducing poverty and achieving sustainable development in developing countries, and improving responses to people with mental illness is an important building block towards achieving this … Unless the needs of people with disability, including those with mental illness, are met, it will not be possible to achieve the targets of the Millennium Development Goals by 2015.^33^

In 2009, the Australian minister for development assistance launched the International Observatory on Mental Health Systems, an initiative of the Centre for International Mental Health.^34^,^35^ The UK Department for International Development has supported two rounds of major funding for Research Program Consortia to “improve [e] mental health services in low income countries.”^36^ Atlantic Philanthropies, a U.S.-based philanthropic organization, has supported two major mental health system development projects in Vietnam, one of which is the National Taskforce for Mental Health System Development in Vietnam.^37^

Mental health has been included among the priority areas for research by the newly established Global Alliance for Chronic Disease,^9^ an initiative that brings together six of the world 's foremost health agencies, which collectively manage an estimated 80% of all public health research funding. Linked with this initiative is the Grand Challenges in Global Mental Health project,^38^ led by the U.S. National Institute of Mental Health, Wellcome Trust, McLaughlin-Rotman Centre for Global Health, and London School of Tropical Medicine. In July 2011, *Nature* published the results of a project setting priorities for global mental health research.^39^ The National Institute of Mental Health has funded three collaborative hubs for international research on mental health and has issued a call for further proposals,^40^ and Grand Challenges Canada has made available a sum of $20 million for Integrated Innovations for Global Mental Health.^38^

There is general agreement that scaling-up activities must be evidence based and that the effectiveness of such activities must be evaluated. If these requirements are to be realized, it will be essential to strengthen capacity in countries to conduct rigorous monitoring and evaluation of system-development projects and to demonstrate sustained benefits to populations. Failure to sustain long-term gains from even well-designed and -implemented community mental health system development projects is a source of serious concern and is all too common.

## CIMH MENTAL HEALTH SYSTEM DEVELOPMENT PROGRAM

**Figure 1:**
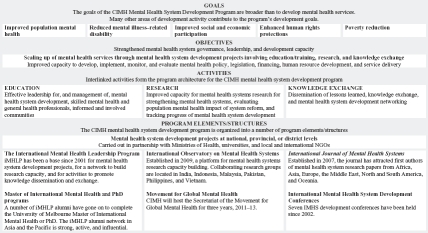
Structure of the CIMH mental health system development program.

The Centre for International Mental Health (CIMH) has been involved in mental health system development work since the centre was established in 1996. This early work demonstrated with great clarity that the key issue was leadership, and supported a developing view that effective mental health system development is not possible without sustained and distributed leadership.^41^,^42^ The CIMH development program (Figure 1) began to assume programmatic coherence in 2001 with the establishment of the International Mental Health Leadership Program (iMHLP), a collaboration between the CIMH, University of Melbourne, and Department of Social Medicine (now Department of Global Health and Social Medicine), Harvard Medical School (in particular, Professor Byron Good and Dr. Alex Cohen).

The goals of the CIMH program are to improve population mental health in LAMICs and in postconflict and post-disaster settings, reduce mental illness–related disability, improve social and economic participation by people with mental disorders, enhance human rights protections for people with mental disorders, and reduce poverty that is related to mental disorders.

## EDUCATION AND TRAINING: iMHLP, MIMH, AND PHD PROGRAMS

The goals of iMHLP,^43^ established in 2001, have remained unchanged: to contribute to the development of effective mental health systems by providing training and mentoring in leadership. The program is aimed at mental health professionals and managers of all disciplines who are in a position to contribute to the development of mental health systems that protect and enhance the human rights of people with mental illness and that are effective, appropriate, accessible, and affordable. The focus of the program is primarily on training leaders for work in developing countries.^44^ iMHLP is a four-week intensive seminar that is followed by mentoring and by supervision of projects in participants' home countries. In addition to formal teaching in leadership skills, the program provides an introduction to mental health policy development and implementation; mental health financing; service design with a focus on community mental health services; human resources for mental health; advocacy and human rights; and mental health systems research. Research- and service-development projects carried out by iMHLP participants are supervised by program faculty and by senior colleagues in the country in which the project is being carried out. During the program's decade of operation, a network of more than 170 alumni has been established across 18 countries in the Asia-Pacific region.

Many of the alumni are now in influential positions in ministries of health, academic departments, WHO, professional associations, and other organizations.

Using iMHLP as a model, the first Master of International Mental Health (MIMH) program was established in 2003. Several iMHLP graduates have undertaken PhD-level studies in the CIMH. iMHLP, MIMH, and PhD alumni are often key collaborators on development and research projects on mental health systems^16^,^18^,^45^–^53^ and in other CIMH development activities, such as the National Task-forces on Mental Health System Development in Indonesia and Vietnam^26^ and the International Observatory on Mental Health Systems.^34^,^35^

## BUILDING RESEARCH CAPACITY

Major advances in pharmacological and other treatments for mental disorders in recent decades have produced few benefits to most of the world 's populations because the mental health systems that are required to deliver such treatments are poorly developed. Whereas high-quality mental health treatment services can be found in the large urban centers in LAMICs, provincial, rural, and remote regions often have no mental health service capacity. In addition to limited service capacity, there is frequently also limited capacity to answer important questions about the mental health status and needs of populations, how mental health is most effectively maintained and illness prevented, whether population wide health policy objectives have been developed and whether they are being achieved, and how health systems can effectively respond to the health needs of the population. High-quality, local research is required to provide the specific, culturally relevant information that is essential for mental health system planning, implementation, monitoring, and evaluation. At present, however, efforts to scale up mental health services^22^ in low-resource environments are hampered by lack of evidence concerning mental health systems, limited capacity to carry out research relevant to policies and practices, and limited capacity among policymakers and health system managers to make use of research findings in decision making. As the then director of the WHO Department of Mental Health and Substance Abuse noted:

It is very important to have good, randomized, clinical trials providing evidence about the efficacy of new treatments but it is equally important to have research providing evidence that a mental health system in a given country, region or district is working better than another. In other words, what we urgently need to know is how to plan and organize services and improve the use of scarce financial and human resources in order to reach out to the mental health needs of the general population and to provide effective and humane services to those who need care.^54^

**Table:** 

Strengthening Mental Health Systems Research Capacity	

Strategy	Notes
Communication infrastructure and active networking	The International Observatory on Mental Health Systems (IOMHS) Secretariat facilitates communication within the IOMHS network, and between IOMHS and other organizations. The networks should be across different institutions and teams, across different parts of the research system (e.g., research producer and user groups), and across different disciplines.
Strategic leadership, training, and skills development	Within countries the IOMHS collaborating groups will contribute to strategic leadership and to shaping the national mental health systems research agenda. Observatory partners are linked into training opportunities offered by CIMH and other organizations, such as the International Master in Mental Health Policy and Services^13^ and the International Diploma in Mental Health Law and Human Rights.^14^
Resource concentration	A central element of the observatory strategy is resource concentration through the establishment of mental health systems research, education, and development groups—which strengthens capacity to secure research funds from national and international sources.
User-researcher interaction	It is essential that the users of the research results are engaged as early as possible in the research process. IOMHS collaborating groups have established links with key research users, particularly ministries of health and civil society and consumer and carer organizations.
Research career development	The establishment of mental health systems research, education, and development groups in key academic departments will open up career pathways that currently do not exist for mental health systems researchers, and will improve ability to compete for scarce research funds. Training will contribute to building careers in mental health systems research.
Ensuring political independence	The establishment of IOMHS collaborating groups in influential academic institutions—which everywhere jealously guard their independence—will contribute to ensuring political independence. However, political independence does not mean political isolation.
Cultural change	A key component of necessary cultural change is the creation of a culture in which the production, dissemination, and use of high-quality research is valued as an integral part of the health system. Knowledge should be publicly owned, widely disseminated, and shared, and should be used for public benefit. Building an open research culture is an essential and long-term objective.

The International Observatory on Mental Health Systems (IOMHS)^34^,^35^,^55^ was inspired by, and modeled on, the European Observatory on Health Systems and Policies,^56^ which “supports and promotes evidence-based health policy-making through comprehensive and rigorous analysis of the dynamics of health care systems in Europe.” The goals of IOMHS are (1) to foster the establishment of, and to support, a network for mental health systems research, education, and development that will produce evidence for mental health policy and practice in LAMICs and that will help build capacity for mental health system development, (2) to produce new knowledge to inform the development of mental health systems that are effective, accessible, equitable, culturally appropriate, affordable, and disability inclusive, and that protect the human rights of people with mental illness, and (3) to monitor and evaluate progress in the development of mental health systems in LAMICs.

IOMHS is a collaborative network of research groups, now focused on Asia and the Pacific, that will measure and track mental health system performance in participating countries at national and subnational (provincial and district) levels. The observatory will build the capability of partner organizations and networks to provide evidence-based advice to policymakers, service planners, and implementers, and will monitor the progress of mental health service scaling-up activities.

The design of the observatory's administrative structures and work programs is based on a strategic approach to strengthening mental health systems research capacity and reflects an effort to ensure that new knowledge is used for the benefit of people with mental illness. The IOMHS's approaches to strengthening research capacity^35^ are outlined in the text box.

## DISSEMINATION AND EXCHANGE OF KNOWLEDGE

### International Journal of Mental Health Systems

The *International Journal of Mental Health Systems* commenced publication in August 2007. It is an open-access, peer-reviewed journal that is part of the stable of Biomed Central independent journals. It has an international editorial board with members from 27 countries—in Africa, North and South America, East and South Asia, Europe, and Oceania—and from many disciplines. The journal is intended as the place to which

mental health system researchers, Health Ministers' advisers, policy makers, mental health consultants advising countries on mental health system development, teachers in psychiatry, nursing, psychology, social work and public health courses, clinicians involved in mental health system reform, and others will turn for the latest research and policy information on how to build equitable, accessible, efficient, high quality mental health systems.^5^

The number of scientific journals and consequently scientific papers devoted to treatment is more abundant than the literature devoted to documenting, analysing and assessing mental health services and mental health system development. The plethora of information on treatment and the prevailing clinical perspective should be gently replaced or, at least, balanced by an effort to bring a public health perspective in mental health. In this sense, it is important to have a journal focusing on mental health system development, which has the capacity of networking good practice in service organization, giving voice to successful experiences including those from low and middle income countries, promoting health services research and mental health services assessment.^54^

The journal has now published over 100 papers, with first authors from every continent. In the difficult, open-access publishing environment, where authors are required to pay substantial article-processing charges, the journal has survived and is growing.

## The International Mental Health System Development Conferences

Seven International Mental Health System Development Conferences were held between 2002 and 2010 in Beijing, Hong Kong, Melbourne, and Taipei. The first and the third were focused on developing leadership and on building capacity in mental health systems research. Each of the other five focused on mental health system development in a specific region or country: Aceh, China, Indonesia, Sri Lanka, and Taiwan. The primary purpose of these conferences is to strengthen the mental health system development network in Asia and to facilitate collaboration. The seventh conference,^57^ held in Melbourne in 2010, is briefly described below as part of the Sri Lanka case example.

## BRINGING IT ALL TOGETHER: SRI LANKA

**Figure 2:**
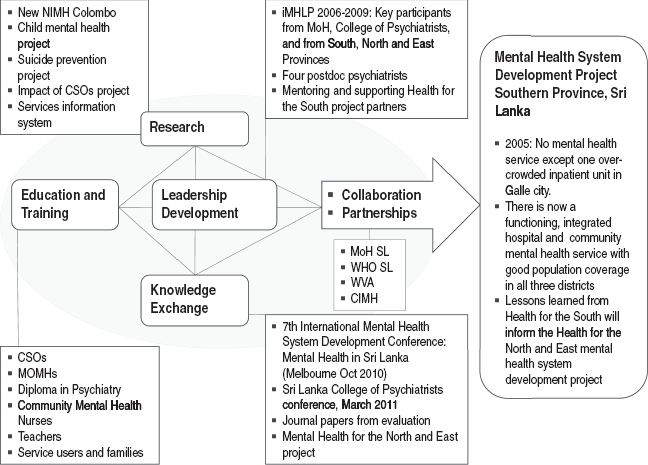
Structure of the CIMH mental health system development program and outcomes of the Health for the South project. CIMH, Centre for International Mental Health; CSO, community service officer; iMHLP, International Mental Health Leadership Program; MoH, Ministry of Healthcare and Nutrition; MOMH, medical officers of mental health; NIMH, National Institute of Mental Health; SL, Sri Lanka; WHO, World Health Organization; WVA, World Vision Australia.

I will use Sri Lanka as a case study to highlight the ways in which the elements of the CIMH program (Figure 1) are integrated for the purpose of mental health system development. The details of the Sri Lanka project and its outcomes can be found in a recently completed evaluation report.^58^ Here I will limit myself to illustrating how the elements of the CIMH program came into play during the course of this project (Figure 2).

In the context of a long-running civil war resulting in massive suffering, death, and displacement over almost three decades, the 2004 Indian Ocean tsunami brought further devastation to the coastal areas of Sri Lanka and resulted directly in 35,000 deaths, many more injuries, and several hundred thousand people displaced from their homes. It fractured communities and destroyed infrastructure and livelihoods. Nearly one million people were affected in 13 districts, including the three districts of the Southern Province—Galle, Matara, and Hambantota. The disaster brought into sharp relief the importance of population mental health and the general absence of mental health services for those in Sri Lanka who needed them.

Many international NGOs, bilateral aid agencies, and UN agencies committed money and technical expertise to a program of reconstruction and recovery following the tsunami. During 2005 and 2006, WHO provided critical support to the Directorate of Mental Health Services of the Sri Lanka Ministry of Healthcare and Nutrition in developing a national mental health policy and implementation plan. As part of this policy, the government of Sri Lanka committed itself to strengthening mental health services, developing a community focus for service delivery, and ensuring that services were accessible to all who needed them.

In 2006, World Vision Australia commissioned an investigation^59^ of the feasibility of developing a model community mental health system in Southern Province, whose population of 2.3 million people was spread across three districts and a large geographical area. One 58-bed, acute psychiatric inpatient unit was available at the Karapitiya General Teaching Hospital in Galle, and two intermediate units were available in Ridiyagama (45 beds) and Unawatuna (40 beds) that housed people with chronic mental disorders for long periods. Virtually no community mental health services were available, and most people with mental illness received no treatment. The investigation 's recommendations were incorporated into the design of the Health for the South Community Mental Health Project (H4S Project).

With funding of $1.1 million over three years, the H4S Project commenced in May 2007 and ended in December 2010. The project goal was to assist the Directorate of Mental Health Services in implementing a pilot project in the Southern Province of Sri Lanka to support the vision of the National Mental Health Policy for Sri Lanka, which was to develop a planned, comprehensive, community-based mental health service organized and implemented through coordination at the national, provincial, district, and community levels, and integrated with general health services at every level of care.

The key partners in the H4S Project were the Directorate of Mental Health of the Ministry of Health, the Provincial and District Directors of Health Services, the WHO Country Office, World Vision Australia, and CIMH. The project was funded by World Vision Australia, authorized and supported by the government of Sri Lanka, and managed by WHO, with CIMH providing technical advice and assistance.

Key senior Sri Lankan health professionals participated in the International Mental Health Leadership Program. They included the national director of mental health services, the provincial director of health services of Southern Province, the Hambantota District director of health services, the president of the Sri Lanka College of Psychiatrists, and two psychiatrists newly appointed to Hambantota and Matara Districts. Four medical doctors have undertaken a yearlong postdoctoral program in CIMH.

Substantial progress has been made in developing the mental health workforce:^58^ establishing a new Diploma in Psychiatry, a one-year training program to address the extreme shortage of psychiatrists in Sri Lanka; training of Medical Officers of Mental Health, who provide the bulk of the primary care–based psychiatric assessment and treatment; training for community mental health nurses; and training and support for community-level mental health volunteers, the Community Service Officers. Training programs have been developed and delivered for large numbers of primary school teachers. Attention has also turned to improving the general community 's level of knowledge about mental health and illness, and, through the creation of consumer and family associations, to encouraging people with mental illness and their families to engage in advocacy for mental health.

Several research projects relevant to policy and practice have been completed, including a WHO Assessment Instrument for Mental Health Systems project,^60^ others focusing on child mental health^61^ and attempted suicide by pesticide poisoning,^62^ and an evaluation of the impact of the Community Service Officers. With support from WHO, the H4S Project, and other donors and partners, the main mental hospital in Colombo has been radically improved and has been designated the Sri Lanka National Institute of Mental Health.

The objective of developing and implementing a model of community mental health services, in line with the National Mental Health Policy of Sri Lanka, has largely been achieved. A comprehensive, community-focused model of mental health services has been established in all three districts of Southern Province. This effort has included the following: acute inpatient units in the district general hospitals of Hambantota and Matara (in addition to the one in Galle); intermediate-care facilities at Unawatuna and Ridyagama (which previously housed long-stay patients with chronic illness but are being transformed into residential rehabilitation facilities); community outreach clinics in most divisions in each district to treat patients in the community; a national toll-free telephone service for mental health advice and counseling; a basic mental health information system in each district; and functioning procedures for intersectoral coordination, including health, social affairs, justice, education, and the police.

The challenges of working in the poorest district in the country and in the context of a continuing civil war were substantial. A number of lessons were learned in the process. The most important were that leadership matters most and that effective mental health system development is possible even in unusually difficult environments.^58^ A year after the completion of the project, the system developments that were accomplished are being sustained and extended by government support.

As a result of active and joint advocacy by World Vision Australia and CIMH, AusAID funded a project focused on developing the mental health system in Sri Lanka's Northern Province. World Vision will “assist the Ministry of Health in the implementation of the community based components of the National Mental Health Policy in the Northern Province through the ‘Reconciliation through integration of Mental Health in Northern Districts’ (REMIND) in Sri Lanka,” a project that will be carried out in collaboration with the Sri Lankan College of Psychiatrists.^63^

The 2010 International Mental Health System Development Conference^64^ focused on mental health development needs in the Northern and Eastern Provinces, which had been devastated by the final phase of the civil war. Among the invited participants were the deputy secretary of the Department of Health, the acting head of the national Directorate of Mental Health, the director of the National Institute of Mental Health, the former mental health adviser in the WHO Country Office, and the head of the AusAID Sri Lanka program. The purpose of the meeting was to review the mental health situation in the Northern and Eastern Provinces and to develop a consensus about how to develop community mental health services. Following the conference, CIMH, the Ministry of Health, and the directors of health services of the Northern and Eastern Provinces made a successful submission to AusAID for the Leadership for Mental Health for the North and East of Sri Lanka project. This project will enable key mental health professionals from Northern and Eastern Provinces to participate in the International Mental Health Leadership Program and to carry out mental health system research and development projects with supervision and mentoring by iMHLP faculty and the Sri Lankan members of the H4S Project.

World Vision Australia's REMIND project and the CIMH project described immediately above are complementary in their objectives, with the first focusing on infrastructure for mental health services and the second focusing on skills and capabilities of key people. Both will be informed by the lessons learned from the H4S Project and will contribute to the development of mental health services both nationally and in the north and east.

## CONCLUSION

I have attempted in this article to give an overview of the CIMH approach to mental health system development. The program architecture no doubt betrays the fact that it has been developed in the context of persistently inadequate resources, shifting and unpredictable opportunities, and grants from various agencies, each with its own objectives and expectations—and few with the flexibility and duration required for system development.

The consistent thread that runs through CIMH's work is the conviction that leadership matters most. Without skilled, sustained leadership at multiple levels—most importantly, from national and local governments—efforts to develop or strengthen mental health systems will fail. And yet, even in what would appear to be the most unpromising of settings, mental health system development has been shown to be possible, even with very modest investment.

In order for such development to succeed, it is necessary to build partnerships that can be sustained over the long haul. The quality of the relationships will be much more important than the specific details of project design. These relationships, like any others, need to be based on such things as honesty, mutual respect, and trust, supplemented by a joint commitment to equity and to protecting the rights of people with mental illness. It is also helpful and even important to enjoy each other's company—which makes it easier to continue working collaboratively when things might not be going so well.

Despite the significant gains that have been made in recent years in putting mental health system development on the general development agenda, there is still a very long way to go. Governments, bilateral and other development agencies, and the large philanthropic organizations are starting to appreciate the importance of population mental health. When the necessary funds begin to flow at a scale that is needed, we need to be ready and to be able to scale up our system-development efforts rapidly and with confidence. The approach that has been presented here has all of the necessary elements and is rapidly scalable.
